# An Information-Theoretic Analysis of Genetics, Gender and Age in Cancer Patients

**DOI:** 10.1371/journal.pone.0001951

**Published:** 2008-04-09

**Authors:** Gurinder Singh Atwal, Raúl Rabadán, Guillermina Lozano, Louise C. Strong, Mariëlle W. G. Ruijs, Marjanka K. Schmidt, Laura J. van't Veer, Heli Nevanlinna, Johanna Tommiska, Kristiina Aittomäki, Gaelle Bougeard, Thierry Frebourg, Arnold J. Levine, Gareth L. Bond

**Affiliations:** 1 The Institute for Advanced Study, Princeton, New Jersey, United States of America; 2 The Cancer Institute of New Jersey, New Brunswick, New Jersey, United States of America; 3 The University of Texas, M.D. Anderson Cancer Center, Houston, Texas, United States of America; 4 Family Cancer Clinic, The Netherlands Cancer Institute, Amsterdam, The Netherlands; 5 Department of Epidemiology, The Netherlands Cancer Institute, Amsterdam, The Netherlands; 6 Department of Clinical Genetics and Human Genetics, VU University Medical Center, Amsterdam, The Netherlands; 7 Department of Obstetrics and Gynaecology, Helsinki University Central Hospital, Helsinki, Finland; 8 Department of Clinical Genetics, Helsinki University Central Hospital, Helsinki, Finland; 9 Inserm U614 and Department of Genetics, Rouen University Hospital, Institute for Biomedical Research, Rouen, France; University of Helsinki, Finland

## Abstract

Germline genetics, gender and hormonal-signaling pathways are all well described modifiers of cancer risk and progression. Although an improved understanding of how germline genetic variants interact with other cancer risk factors may allow better prevention and treatment of human cancer, measuring and quantifying these interactions is challenging. In other areas of research, Information Theory has been used to quantitatively describe similar multivariate interactions. We implemented a novel information-theoretic analysis to measure the joint effect of a high frequency germline genetic variant of the p53 tumor suppressor pathway (MDM2 SNP309 T/G) and gender on clinical cancer phenotypes. This analysis quantitatively describes synergistic interactions among gender, the MDM2 SNP309 locus, and the age of onset of tumorigenesis in p53 mutation carriers. These results offer a molecular and genetic basis for the observed sexual dimorphism of cancer risk in p53 mutation carriers and a model is proposed that suggests a novel cancer prevention strategy for p53 mutation carriers.

## Introduction

The p53 stress response pathway functions as a critical tumor suppressor pathway, as demonstrated by the observation that the p53 gene is one of the most commonly mutated genes in human tumors [1]. Furthermore, both mice and humans harboring a germ-line inactivating mutation in just one allele of the p53 gene develop tumors early in life and at very high frequencies [2]-[5]. Humans harboring a germ-line inactivating p53 mutation make up 50% of the members of the Li-Fraumeni cancer predisposition syndrome. The age of onset of tumor formation in human p53 mutation carriers has been shown to be modified in four independent studies by a high frequency single nucleotide polymorphism in the promoter of the Mdm2 oncogene (MDM2 SNP309, T/G) [6]-[9]. The G-allele of SNP309 increases the DNA binding affinity of the transcriptional activator Sp1, which results in higher levels of MDM2 mRNA and protein in human cells and tissues [6], [10]-[12]. Higher levels of MDM2 lead to the attenuation of the p53 pathway, in concordance with the role of MDM2 as a key negative regulator of p53 [13]. In p53 mutation carriers, it was shown in three independent reports that individuals with the G-allele of SNP309 are diagnosed with cancer on average seven to fifteen years earlier than those p53 mutation carriers homozygous for the T-allele (Table 1) [6]-[8]. It was proposed that the high levels of MDM2 resulting from the G-allele of SNP309, together with the mutant p53 allele, produce a severely weakened p53 tumor suppressor pathway and result in a higher mutation rate, poorer DNA repair processes, and reduced apoptosis, leading to faster and more frequent tumor formation [14].

**Table 1:** Characteristics of three patient populations used to define MDM2 SNP309 as a modifier of tumorgenisis in p53-mutation carriers.

		Number of Affected Carriers	Earlier Average Age of Tumor Diagnosis for G-allele Carriers (years, reported p-value)	Orignal Reference	Number of Carriers with a Cancer other than Breast, Ovarian or Prostate	Number of Females	Number of Males
p53 Mutation Carriers	M.D. Anderson Cancer Center	66	7 (p = 0.031)	Bond, Wu et al., 2004	48	18	30
	French LFS Network	42	10.3 (p<0.05)	Bougeard et al., 2006	29	16	13
	Dutch/Finnish Study	36	15.8 (p = 0.005)	Ruijs et al., 2006	24	10	14

Recently, studies with sporadic cancers (162 Ashkenazi Jewish lymphoma patients, 969 Finnish and 164 Italian colorectal cancer patients, 105 German sarcoma patients, 341 Norwegian non-small cell lung cancer patients and 658 Ashkenazi Jewish breast cancer patients) have demonstrated that the effects of the G-allele of MDM2 SNP309 locus on tumorigenesis can be modified by two additional variables; namely gender and the primarily female-specific hormone, estrogen [15]-[19]. Specifically, the G-allele of MDM2 SNP309 was shown to accelerate tumorigenesis and increase cancer risk in women and not in men for colorectal cancer, diffuse large B-cell lymphoma, lung cancer and for highly estrogen receptor positive (>50% of tumor cells), but not for estrogen receptor negative, invasive ductal carcinoma of the breast[16], [18], [19]. This was shown to result in the enrichment of individuals with the G-allele in pre-menopausal women with these cancers, when compared to either post-menopausal women or men with the same cancers. Recently, Hu et al. provided evidence for a possible molecular mechanism for how the G-allele of SNP309 could accelerate tumor formation in this gender-specific and estrogen dependent manner, by demonstrating that the primarily female-specific hormone, estrogen, preferentially stimulated transcription of the MDM2 gene with the G-allele of SNP309 [20].

Interestingly, two independent studies have also defined gender to be a modifying factor of cancer risk in p53 mutation carriers [21], [22]. Specifically, female p53 mutation carriers were shown to be at greater risk for developing cancer than their male counterparts. For example, by 20, 30, 40, and 50 years of age, the female carriers showed respective cumulative risks of 18%, 49%, 77% and 93% for developing a first cancer, while the male carriers showed risks of 10%, 21%, 33% and 68% [22]. The increased risk in female carriers could not be explained by the high incidence of gender-specific cancers, as the differences remained after the exclusion of cases of breast, ovarian, and prostate cancers. Although the possible involvement of estrogen in increasing cancer risk in female carriers was discussed, this hypothesis remains untested.

It is intriguing to hypothesize that the SNP309 locus could contribute to the increased cancer risk observed in female carriers though the preferential estrogen-dependent stimulation of MDM2 transcription from the G-allele of SNP309. If true, estrogen manipulation could be incorporated in prevention strategies for p53-mutation carriers with the G-allele of MDM2 SNP309. Such patients have limited cancer prevention strategies. In this report, the hypothesis that the primarily female specific hormone, estrogen, could affect tumorigenesis in p53-mutation carriers is tested though exploring possible interactions between MDM2 SNP309 locus and gender upon the age-dependent tumor incidence.

There has been increasing interest in the development of statistical tools to uncover such interactions, as between genotype and gender, and their effect upon complex phenotypes, such as age-dependent tumor incidence. Recent methods, both parametric and non-parametric, include neural networks [23], recursive partitioning methods [24], combinatorial partitioning methods [25], multifactor-dimensionality reduction [26] and multi-locus regression [27], [28]. These methods are tailored to address specific questions about the nature of multi-locus interactions and invoke different assumptions about the underlying model generating these data. Here we employ a novel information-theoretic framework to address the specific question of whether gender and the MDM2 SNP309 locus interact synergistically or redundantly with respect to the age of tumor incidence in p53 mutation carriers. The optimal properties of information-theoretic terms in assessing variability and correlation between multiple variables are familiar to the machine learning and statistics communities but perhaps not well known in fields of epidemiology and medical statistics. An additional advantage in the context of this present study is that the principles of information theory allow us to study complex phenotypes expressed as real numbers (i.e. age of onset of cancer as opposed to the binary case/control scenario) by defining the optimal partitioning, or binning, of the continuous phenotype data.

## Methods

### p53 Mutation Carriers (M.D. Anderson Cancer Center)

88 individuals who are members of Li-Fraumeni syndrome (LFS) families (34 total) with known germline mutations in one allele of p53 were previously genotyped for MDM2 SNP309 [6]. 66 of these have been diagnosed with at least one cancer. Of the LFS families included in this study, 12 were ascertained through systematic studies of sequential childhood soft tissue sarcoma (7 families) and osteosarcoma (5 families) patients. The remaining families consisted of patients referred to M. D. Anderson for research or clinical studies with a personal or family history of cancers of the type found in LFS. The largest family contributes 14 samples to this study; 16 families contribute a single individual.

### p53 Mutation Carriers (French LFS Network)

Sixty-one affected or unaffected germ-line p53 mutation carriers, from 41 unrelated LFS families were previously genotyped for MDM2 SNP309 [7]. Clinical characteristics of the families and description of the mutations were available upon request. Forty-one affected germ-line p53 mutation carriers were incorporated into this study. The largest family contributes 3 affected individuals to this study; 27 families contribute a single affected individual.

### p53 Mutation Carriers (Dutch/Finnish Study)

One hundred and eight patients from 87 LFS or LFS-like families were previously screened for p53 mutations and genotyped for MDM2 SNP309 [8]. Ninety-seven were screened at the Family Cancer Clinic of the Netherlands Cancer Institute and 11 at the Helsinki University Central Hospital. Thirty-six affected individuals from 19 families carried known germline mutations in the p53 gene and were incorporated into this study.

### Information-Theoretic Analysis

Given a variable, say *X*, which can exist in one of *K_X_* states, and its set of associated probabilities {*p(X = x_1_),p(X = x_2_)…p(X = x_K_)*}, then Shannon's entropy 
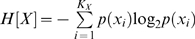
 provides a general non-parametric measure of the variability, or information content, of *X*. This measure of information content is the only quantity that satisfies certain reasonable criteria such as *H[X]* ought to be an increasing function of *K_X_* if all the probabilities are equal. The joint entropy for two variables measured together,say *X* and *Y*, can also be appropriately defined, 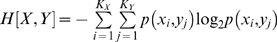 where *p*(*x_i_*,*y_j_*) is the probability for the two events *X = x_i_* and *Y = y_j_* co-occurring. It can be shown that the following inequality for the joint entropy must always apply, *H*[*X*,*Y*]≤*H*[*X*]+*H*[*Y*], with equality if and only if *X* and *Y* are independent variables *p*(*x_i_*,*y_j_*) = *p*(*x_i_*)*p*(*y_j_*), i.e. the joint entropy will always be less than the sum of the individual entropies if there is any dependence, or correlation, between the two variables. The measure of statistical dependency between two variables, *X* and *Y*, is then simply captured by the reduction in total entropy when the two variables are measured together, i.e. Shannon's mutual information 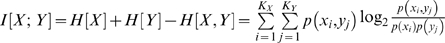. Note that this does not invoke any of the linear (Gaussian) assumptions required by other measures such as Pearson's correlation. This definition can also be equivalently written as *I*[*X*;*Y*] = *H*[*X*]−*H*[*X*∥*Y*] = *H*[*Y*]−*H*[*Y*∥*X*], where the conditional entropy *H*[*X*∥*Y*] is defined as 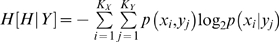, making it evident that *I[X;Y]* can be interpreted as the reduction of the variability of *X* due to knowledge of *Y*, and vice versa.

Data analysis using discrete versions of information-theoretic expressions, as defined in the previous paragraph, requires creating bins that discretize continuous values. For example, the real number representing the age of diagnosis of cancer has to binned into one of several groups, essentially a task in data compression. We can draw upon information theory and note that the least biased partitioning of any dataset is given by the principle of maximum entropy, i.e., partitioning into equally populated bins or quantiles. In this case any information-theoretic expression calculated from the binned data has the important and desirable property that it is invariant to invertible coordinate transformations, e.g. multiplying all the ages by a constant factor should not affect the mutual information with the SNP variable. A common problem with quantizing real numbers into bins is deciding how many bins to use, and to this end we again appeal to information theory. A sensible criterion can be gleaned from the observation that if we try to measure the mutual information between two finitely-sampled independent distributions then employing too many bins will over-fit the data, giving rise to a non-zero value of mutual information, even when extrapolated to the infinite sample limit [29]. On the other hand, compressing the data into too few bins over-smoothes the data, and potentially loses important structure in these data. Thus there ought to be an optimal trade-off between over-fitting and information compression. We determined that the optimal number of bins in our dataset were three, and to establish this, the mutual information values were first calculated with randomly shuffled data, to destroy any correlation, and it was observed that when the number of bins was increased from three to four and higher, the extrapolated mutual information values was no longer zero within error bars. Thus by restricting the number of bins we implicitly incorporate a sample-size dependent penalty for over-fitting of the data. Further explicit details on this and other possible methods of estimating mutual information are given in [29], [30]

### Synergistic Information

These information-theoretic concepts can be extended to naturally probe questions of statistical dependencies amongst more than two variables. In particular, levels of synergy and redundancy amongst three variables can be quantified in a principled manner, and have recently been formulated in the context of analyzing correlations between neurons [29], [30]. If we use the definition of synergy between two variables *X,Y* with respect to a third variable *Z*, as how much extra information *X* and *Y* together provide about *Z* than by *X* and *Y* separately, then we are immediately lead to the natural formulation of synergistic information as *S(X,Y;Z) = I(X,Y;Z)-I(X;Z)-I(Y;Z)* where 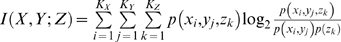. The synergistic information thus reveals how much of the variability of *Z* is reduced by knowing both *X* and *Y* together than knowing them separately and thus rigorously quantifies the non-independent effects of both *X* and *Y* on *Z*. This mathematical formulation of synergy can be generalized naturally to more than three variables.

### Statistical Analysis

It may not be clear how to accurately interpret information-theoretic values determined from empirical distributions, especially as statistical fluctuations grow with decreasing sample size. Furthermore, due to the concavity of Shannon's entropy, finite sampling induces a negative bias in the empirical entropy and a positive bias in the empirical mutual information [29]. These problems necessitate generation of p-values to the calculated information-theoretic values against a specified null hypothesis. In the case of mutual information, the null hypothesis was that no statistical dependence existed between a single variable (either SNP or gender) and age of diagnosis, i.e., the joint probability is simply a product of the marginals, *p(variable,age) = p(variable)p(age)*. In the case of synergistic information, the null hypothesis was that no correlation existed between two variables and age of diagnosis while preserving the correlation between the two variables, i.e. *p(SNP,gender,age) = p(SNP,gender)p(age)*, *p(SNP,age) = p(SNP)p(age)* and *p(gender,age) = p(gender)p(age)*. The p-values were determined by a Monte Carlo permutation test. A minimum number of 10^6^ permutations were required to generate a confidence interval range of less than 0.005 at p-value 0.05.

A two-sided Mann-Whitney test was employed to determine the statistical significance of the difference in the median age of tumor diagnosis between the groups with and without the G-allele of SNP309. Strictly speaking, the Mann-Whitney test is a non-parametric assessment of whether two sets of data are drawn from the same distribution, but if the two sets of data have roughly the same shape then it is justified to interpret the results of the test as a statement on whether the median in the two groups are statistically not different. A permutation test was employed to determine the significance of the difference in the average age of tumor diagnosis between the two groups [6]. Both a Kolmogorov-Smirnov test and a Cramer-Smirnov-Von Mises test were utilized to determine the significance of the difference in the distributions of age of diagnosis between the groups with and without the G-allele of SNP309. By employing all of these tests for significance, many of the assumptions inherent in any one test are eliminated.

## Results

To test the effects of, and the interactions between, gender and the alleles at the MDM2 SNP309 locus gene upon tumorigenesis in p53 mutation carriers, all 144 p53 mutation carriers, who were genotyped at the MDM2 SNP309 locus previously [6]-[8], were studied (Table 1). Collectively, when the 144 individuals were separated into the different genotypes of the SNP309 locus, individuals who carried the G-allele in either the heterozygous or homozygous state (n = 81), showed a very significant 11-year earlier average age of first tumor onset compared to individuals T/T (n = 63) in genotype (T/T individuals, average is 33 years of age versus 22 years of age in G/G and T/G individuals, p = 0.0006, Mann Whitney Test). A significant earlier average age of first tumor onset among G-allele carriers was also seen in both females and males. Males who carried the G-allele in either the heterozygous or homozygous state (n = 35), showed a significant 12-year earlier average age of first tumor onset compared to males T/T (n = 23) in genotype (p = 0.0436, Mann Whitney Test). Females who carried the G-allele in either the heterozygous or homozygous state (n = 46), showed a significant 10-year earlier average age of first tumor onset compared to females T/T (n = 40) in genotype (p = 0.0223, Mann Whitney Test).

**Figure 1:**
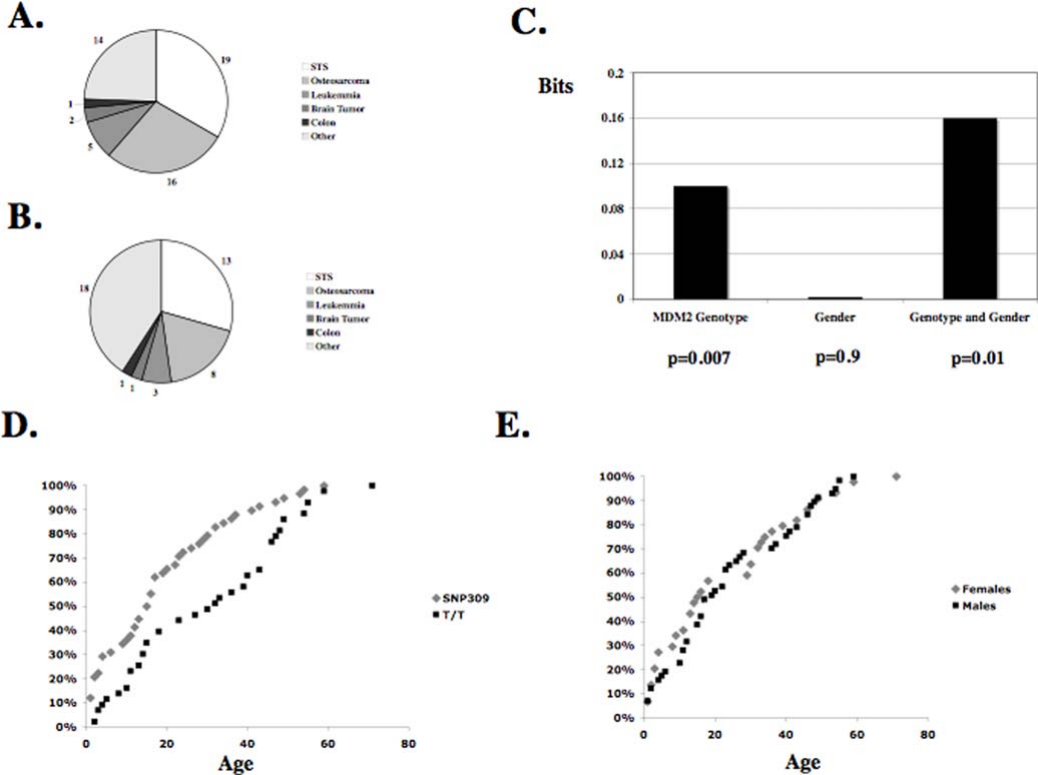
An information-theoretic analysis reveals a significant level of mutual information between the MDM2 SNP309 locus, but not gender, and the age of tumor onset. In order to compare the age dependent incidence of cancer between males and females, individuals who developed primarily gender-specific cancers were excluded from this analysis, leaving STS, osteosarcomas and leukemmias as the major tumor types analyzed as depicted in the pie charts for both males (A) and females (B). (C) The bar graph depicts the bits of mutual information between the MDM2 SNP309 locus and the age of tumor onset, gender and the age of tumor onset and lastly between all three variables. The associated p-values are depicted below. (D) The cumulative incidence of tumor diagnosis for both the individuals T/T in genotype (black squares) and T/G or G/G (SNP309) in genotype (grey diamonds) is plotted as a function of age. A square or a diamond represents at least one individual. (E) The cumulative incidence of tumor diagnosis for both males (black squares) and females (grey diamonds) is plotted as a function of age.

In order to directly compare the age dependent incidence of cancer between males and females, individuals who developed primarily gender-specific cancers (breast and prostate cancer) were excluded from this analysis, leaving STS, osteosarcomas and leukemias as the major tumor types analyzed in both genders (Figure 1A and B). Of the remaining 101 individuals, 57 individuals were male and 44 were female (Table 1). The mutual information shared between gender, the G and T alleles of MDM2 SNP309 and the age of first tumor diagnosis were calculated and the results are presented in Figure 1C. As expected from the previously published analyses of these populations [6]-[8], the MDM2 SNP309 locus affects the age of tumor diagnosis in a significant manner as demonstrated by a significant level of mutual information (0.10 bits, p = 0.007, Permutation Test). In contrast to what was expected from the previous assessments of overall cancer risk in p53 mutation carriers [21], [22], gender alone did not show a significant level of mutual information with the age of first tumor diagnosis (0.002 bits, p = 0.9, Permutation Test). Indeed, when the 101 individuals were separated into the different genotypes of the SNP309 locus, individuals who carried the G-allele in either the heterozygous or homozygous state, showed a very significant 12-year earlier average age, and 17-year median age of first tumor onset compared to individuals T/T in genotype (T/T individuals, average is 30 years of age, ranging 1 to 71 years, versus 18 years of age in G/G and T/G individuals, ranging from 1 to 59 years, Figure 1D and Table 2). Statistical significance of this 12-year earlier average age, and 17-year earlier median age of tumor onset was measured using a permutation test and a Mann-Whitney test (see Materials and Methods, p = 0.0008 and p = 0.0015, Table 2). Furthermore, the significance of the difference in the distributions of the ages of tumor diagnosis between G-allele carriers and individuals T/T in genotype, as seen in Figure 1D, was measured using two approaches: the Kolmogorv-Smirnov Test and the Cramer-Smirnov-Von Mises Test. Both tests supported the significance of these differences by yielding p-values 0.0091 and 0.0046, respectively (Table 2). In contrast, when the 101 individuals were separated based on gender alone, no significant differences in the average age of tumor onset were seen. Specifically, female p53-mutation carriers were diagnosed on average at 23 years of age (ranging from 1 to 71 years of age) compared to the male carriers who were diagnosed on average at 24 years of age (ranging from 1 to 59 years of age, Figure 1E and Table 2).

**Table 2:** The effect of the MDM2 SNP309 locus and gender on the age of diagnosis in p53 mutation carriers.

		Number	Average Age	Median Age	Permutation Test for Difference in Average (p-value)	Mann-Whitney Test (p-value)	Kolmogorv-Smirnov Test (p-value)	Cramer-Smirnov-Von Mises Test (p-value)
MDM2 SNP309 Locus	T/T	43	30	32	0.0008	0.0015	0.0091	0.0046
	SNP309	58	18	15				
Gender	Males	57	24	19	0.7468	0.5216	0.3348	0.3989
	Females	44	23	15				
Males	T/T	22	30	33	0.0285	0.0672	0.0478	0.0469
	SNP309	35	20	17				
Females	T/T	21	31	32	0.007	0.0037	0.0275	0.0077
	SNP309	23	15	9				

**Figure 2:**
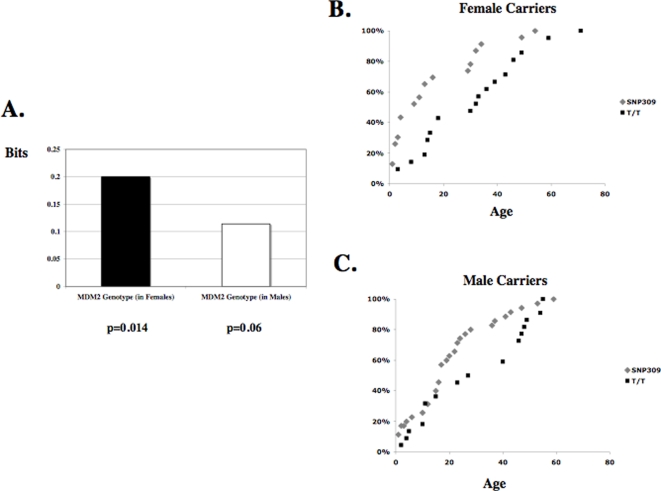
The G-Allele of SNP309 Associates with an Accelerated Age of Diagnosis of Non-Gender Specific Tumors in Female but not in Male p53 Mutation Carriers. (A) The bar graph depicts the bits of mutual information between the MDM2 SNP309 locus and the age of tumor onset in females and in males. The associated p-values are depicted below. The MDM2 SNP309 locus and the age of tumor diagnosis share significant mutual information only in females and not in males. Statistical significance is represented by the black labeling of the bar graph and the p-values depicted below. The cumulative incidence of all non-gender specific tumor types for both the individuals T/T in genotype (black squares) and T/G or G/G in genotype (grey diamonds) is plotted as a function of age for females (B) and males (C). A square or a diamond represents at least one individual.

As mentioned above, one advantage of an information-theoretic analysis of interactions is that non-additive interactions can be detected and quantified. Interestingly, this analysis revealed that the MDM2 SNP309 locus and gender taken together interact to affect the age of tumor diagnosis in a non-additive, synergistic manner, as demonstrated by the measured 0.06 bits of significant synergistic information (p = 0.05, Permutation Test). This suggests that the well-defined modifying effect of the MDM2 SNP309 locus upon the age of first tumor onset in p53-mutation carriers (Table 1) could be significantly different between the genders. To test this, the interaction of the alleles of MDM2 SNP309 with the age of tumor diagnosis was measured for both genders by calculating their respective mutual informations. Interestingly, by this measure, the MDM2 SNP309 locus only significantly interacts with the age of tumor diagnosis in female carriers and not in male carriers. Specifically, in female carriers, the MDM2 SNP309 locus and the age of tumor diagnosis share a significant 0.2 bits of mutual information (p = 0.014, Figure 2A). However, in male carriers, the 0.114 bits of mutual information measured does not reach significance with a p-value of 0.06.

When separated into the different genotypes of the SNP309 locus, the 23 females who carried the G-allele in either the heterozygous or homozygous state, show a significant 16-year earlier average age, and 23-year median age of tumor onset compared to the 21 females T/T in genotype (T/T females, 31 years of age, ranging 2 to 71 years, versus 15 years of age in G/G and T/G females, ranging from 1 to 54 years, p = 0.007 and p = 0.0037, Figure 2B and Table 2). Their respective distributions of the ages of tumor diagnosis also significantly differ, as measured by the Kolmogorov-Smirnov Test and Cramer-Smirnov-Von Mises Test (p = 0.0275 and 0.0077, Table 2). As predicted by the information-theoretic analysis the differences between the male G-allele carriers and the males T/T in genotype are much weaker. When separated into the different genotypes of the SNP309 locus, males who carried the G-allele in either the heterozygous or homozygous state, showed a significant 10-year earlier average age of tumor onset compared to males T/T in genotype (T/T males, 30 years of age, ranging 1 to 55 years, versus 20 years of age in G/G and T/G females, ranging from 1 to 54 years, p = 0.0285, Figure 2C and Table 2). However, the measured significance of the differences in the medians and distributions in males were just boarder-line significant with p-values of 0.0672, 0.0478 and 0.0469 respectively (Table 2). Thus the p-values calculated in several different ways were 5-10 fold weaker for the impact of the G-allele upon the age of onset of first tumors in males than females.

## Discussion

In this report, the statistical dependencies of the MDM2 SNP309 locus and gender upon the age of first tumor onset in p53 mutation carriers were determined using a novel quantitative information-theoretic approach. The three patient populations that were originally used to define MDM2 SNP309 as a modifier of tumorigenesis in p53 mutation carriers were analyzed (Table 1). The analysis revealed a significant level of mutual information between the MDM2 SNP309 locus and the age of first tumor onset, but not between gender and the age of tumor onset (Figure 1). The information-theoretic approach allowed for the probing of statistical dependencies among all three variables (the alleles of MDM2 SNP309, gender and the age of first tumor onset). Interestingly, the MDM2 SNP309 locus and gender taken together were shown to interact and affect the age of non-gender specific tumor diagnosis in a non-additive, synergistic manner (Synergistic Information = 0.06 bits; p = 0.05). In females, the MDM2 SNP309 locus and the age of tumor diagnosis share significant mutual information whereas, in males, the level of mutual information measured did not quite reach significance (Figure 2). In other words, the analysis of the three variables in this study revealed that gender does in fact play a role in age-dependent incidence but only when information is also provided about the allelic form of the MDM2 SNP309 locus. This is likely due to the observation that only the G-allele at MDM2 SNP309 is stimulated to produce more MDM2 mRNA and protein in the presence of estrogen [20]. Standard statistical tests measuring the significance of the differences both in the mean and median ages of first tumor onset and in the distributions of the ages of tumor diagnosis between G-allele carriers and individuals T/T in genotype validated these observations (Table 2).

As mentioned above, the information-theoretic approach can probe questions of statistical dependencies among more than two variables and can quantify levels of synergy and redundancy. Large-scale genetic and epidemiological studies increasingly have acknowledged that variables interact in a non-additive manner with respect to risk of disease, i.e. the variables do not act independently of each other. Highlighting these dependencies is critical in understanding how disease risk can be modified subtly by a hidden variable and can explain why different epidemiological studies can report different conclusions based on viewing a single variable [17]. Furthermore, the identification and quantification of synergistic interactions also can shine a light on new biological mechanisms, such as cross talk between two signaling pathways. However, as yet, there has been no rigorous definition and thus, no quantification of such synergistic interactions. Information theory and the methods detailed here form a natural framework to address these issues of statistical dependencies among variables while invoking no parametric assumptions.

In conclusion, the information-theoretic analysis of the statistical dependencies of the MDM2 SNP309 locus and gender upon the age of tumor onset in p53 mutation carriers suggests that the G-allele of SNP309 functions primarily in female p53 mutation carriers to accelerate tumor formation, thereby providing a potential genetic basis for observed sexual dimorphism in cancer risk [21], [22]. These observations in conjunction with the observations that both pre-pubescent and post-pubescent females have higher levels of estrogen [33], [34], and that estrogen has been shown to preferentially stimulate the transcription of *MDM2* from the G-allele [20], provide a reasonable hypothesis; the G-allele of the MDM2 SNP309 locus could contribute to the increased cancer risk observed in female p53 mutation carriers through the preferential estrogen-dependent stimulation of transcription of the MDM2 gene. If true, this model predicts that estrogen reduction or withdrawal from G-allele MDM2 SNP309 patients could be incorporated in prevention strategies for p53-germlinc carriers, for whom cancer prevention strategies are limited, much as they have been successfully implemented for BRCA-mutation carriers [35]-[37].
